# Paediatric Electrophysiologic Studies: How and What With?

**Published:** 2008-05-01

**Authors:** N Sreeram, M Emmel, U Trieschmann, E de Haan

**Affiliations:** University Hospital of Cologne, Germany

**Keywords:** pediatric, electrophysiologic studies

## Abstract

Catheter ablation of arrhythmias in children has become standard practice virtually worldwide. Successful and safe ablation has been made possible by a combination of factors. These include increased operator experience, a better understanding of the natural history of a wide variety of arrhythmias, advances in technology such as smaller catheters, the routine use of various three-dimensional mapping systems, and the development of alternative energy sources. It is also not uncommon to perform multiple catheter intervention procedures (ablation ± intravascular stent implantation ± device closure of residual shunts ± elective pacemaker or device implantation) during a single session. It is important to bear in mind that arrhythmia recurrence is commoner in children in general, and that this is particularly the case with postoperative (scar-related arrhythmias). Despite acute success, long-term follow-up is mandated for this subgroup of patients.

## Introduction

Invasive electrophysiologic studies in children have derived from adult practice, but are tailored to the special needs for infants and children. Such differences between children and adults relate to several issues. These include the smaller size of the patient (and therefore the heart and the vessels used for access), the need for general anaesthesia when performing EP studies of prolonged duration, and the different spectrum of diseases encountered in paediatric EP practice. While a significant proportion of paediatric ablation procedures are for arrhythmia substrates commonly seen in adult practice also (accessory pathways, AV nodal reentry, ectopic foci or reentrant circuits in the atria and ventricles), an important and growing distinction between the two is in the field of post-surgical (usually scar-related) arrhythmias following successful surgical repair of complex congenital cardiac malformations. Successful therapeutic strategies for these arrhythmia substrates demands a thorough knowledge of the anatomy of the original lesion, the alterations in the intracardiac connections produced by surgical repair including the location of atriotomy scars, synthetic baffles and suture lines, and the natural (and post-surgical) history of some of the commoner lesions contributing to late postoperative arrhythmias.

## Elective ablation in children

Elective catheter ablation of common arrhythmia substrates in children can usually be safely undertaken from around 4 years of age and upwards. The majority of arrhythmias in this age group are associated with atrioventricular accessory pathways [1-3]. It is well recognized that such reentrant atrioventricular arrhythmias show a bi-modal age distribution, with a lot of symptomatic infants having no further arrhythmias between the ages of 1 and 5 years [4]. Recurrence of clinical arrhythmia beyond 5 years of age usually predicts further recurrences into adult life. Spontaneous cures of accessory-pathway related arrhythmias must therefore be uncommon, and reflects the lack of adequate serial long-term follow-up of individual patients. It is not entirely surprising that when RF ablation first came into clinical practice in the adult population, the majority of arrhythmia substrates undergoing ablation were atrioventricular accessory pathways and AV nodal reentry tachycardia!

Ablation under 4 years of age, and particularly in infancy, can also be safely performed. The indications for ablation however need to be carefully evaluated and weighed against the potential risk of catheter-related complications (which have include death in the early series, complete heart block requiring a permanent pacemaker, thrombotic and vascular complications, and coronary arterial spasm, stenoses or occlusion). Ablation in this age group is therefore generally reserved for incessant or potentially life-threatening arrhythmias, which are resistant to pharmacologic therapy (an arbitrary definition), are associated with haemodynamic compromise (some post-operative arrhythmias), or with tachycardia-induced cardiomyopathy [5-7].

### Example 1

**Figure 1:**
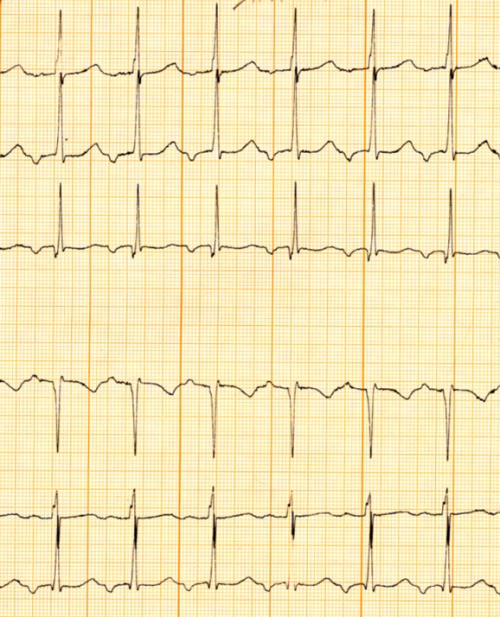

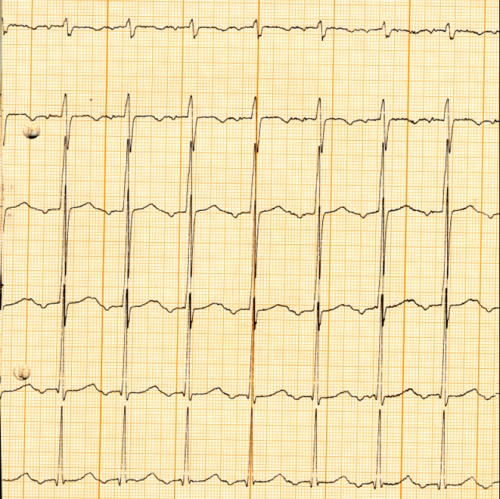
***a and b:*** Persistent junctional reciprocating tachycardia in a young patient, who had tachycardia-associated cardiomyopathy. This is one of the few indications for performing catheter ablation in infancy.

**Figure 2:**
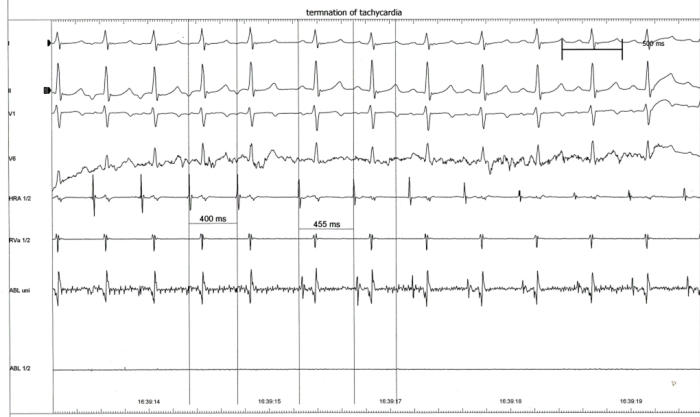
Intracardiac electrogram during ablation, showing termination of tachycardia. Tachycardia terminates after the 5th QRS complex, by retrograde block in the pathway, with restoration of sinus rhythm. This was followed by rapid recovery of cardiac function.

This patient presented in infancy with incessant tachyarrhythmia, mediated via a posteroseptal accessory pathway with decremental properties (persistent junctional reciprocating tachycardia) (Figure 1a and b). In view of difficulty in controlling the arrhythmia with medications, and tachyarrhythmia-related dilated cardiomyopathy, elective catheter ablation was performed at 2 years of age (Figure 2). Successful ablation was associated with complete recovery of myocardial function.

## General anaesthesia

The majority of EP studies in children under 10 years of age require general anaesthesia for smooth conduct of the study. This in turn mandates a well equipped EP lab, with experienced anaesthesiologists and support staff. Occasionally, specific arrhythmias (especially automatic tachycardias) may be non-inducible under anaesthesia, and require that the degree of anaesthesia be progressively lightened until the patient is almost awake. Where such measures also fail, there may be no other option but to redo the study under conscious sedation. Occasionally, in patients with a previously documented narrow QRS tachyarrhythmia and demonstrable dual AV nodal physiology or sustained slow pathway conduction during EP study, we have opted to modify the AV node (slow pathway ablation), even in the absence of inducible arrhythmia in the catheter laboratory [8,;9].

It cannot be overstressed that EP studies in patients with complex anatomy should be performed in a laboratory equipped with pressure recording and angiographic facilities. Presently EP studies are often combined with either diagnostic catheterization (for pre- or post-surgical evaluation of structural cardiac defects). Increasingly however, it is also the case that ablations are combined with interventional therapeutic procedures (closure of congenital intracardiac shunts, surgical fenestrations, stent and pacemaker implantation). With some of these procedures, access to certain cardiac chambers may no longer be possible following either surgery (for example, a Fontan completion using an extracardiac conduit), or device closure of an interatrial defect (for example, fenestration closure of an interatrial communication after the Fontan procedure). It is important therefore that every opportunity is taken to ablate arrhythmogenic substrates prior to such definitive surgical or catheter-based interventions. Equally, it is advantageous for the patient that such combined procedures are conducted during a single session under anaesthesia.

## Vascular access and catheter introduction

It is not uncommon that EP studies require the introduction of between 3 and 5 electrode catheters in a child. The majority of standard electrode catheters are in the range of 5 to 8 French in size, and are therefore unsuitable for small children. This problem may be addressed in one of two ways: the first, and most practiced, is to use multiple entry sites in order to avoid vessel damage or thrombosis. This means that the EP study is often performed using combined femoral, jugular and occasionally subclavian or even transhepatic vascular access [10,11].

The second approach has been to use microcatheter technology. Some of these catheters, which are as small as 1.7 F in diameter, are of particular value in cannulating the coronary sinus, especially when mapping accessory pathways which are located very anteriorly on the mitral valve annulus [12]. They are also invaluable for delineating the right atrioventricular groove in children with Ebstein's anomaly which is associated with apical displacement of the septal and posterior leaflets of the tricuspid valve, and in whom multiple right sided accessory pathways are often present.

### Example 2

**Figure 3:**
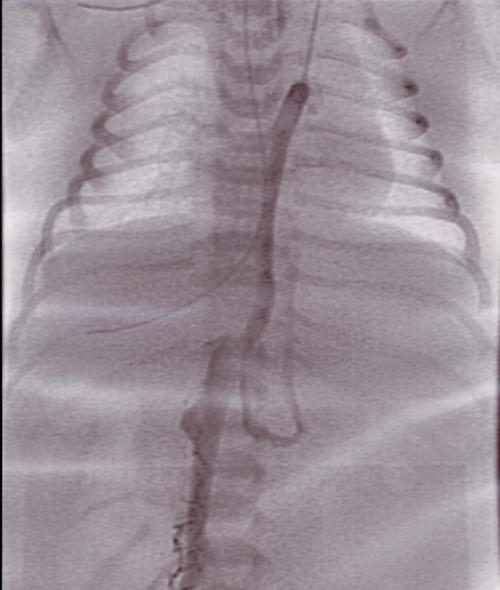
Femoral venous angiogram demonstrating azygos continuation in an infant with visceral heterotaxy and recurrent SVT.

**Figure 4:**
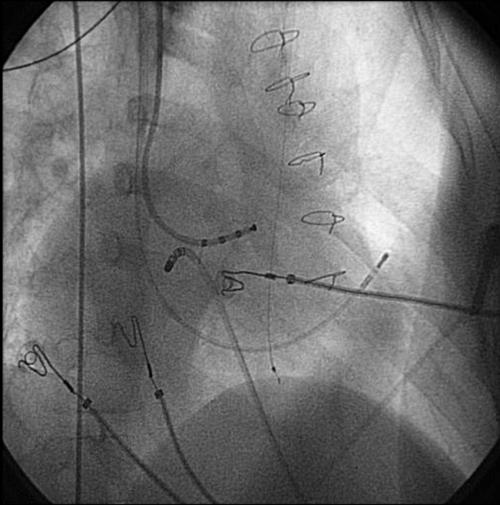
Catheter positions (the ablation catheter approaches the AV groove via a left sided liver vein) in another young patient with visceral heterotaxy, undergoing ablation of an accessory pathway.

**Figure 5:**
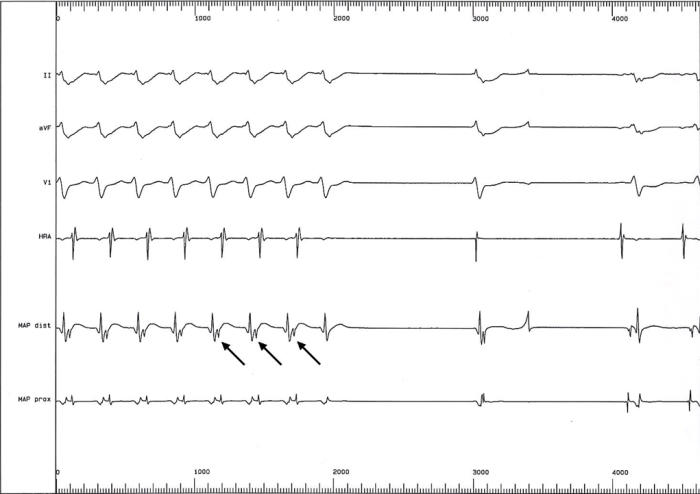
Termination of tachycardia by retrograde block in the patient whose angiogram is shown in figure 3. Successive retrograde p waves are shown by arrows.

This infant with complex structural heart disease, visceral heterotaxy, and absent inferior vena cava with azygos continuation (Figure 3) presented with a recurrent reentrant supraventricular tachycardia. Access to the AV groove was achieved by direct puncture of the hepatic veins (which were in a mirror-image arrangement compared with normal situs). Figure 4 shows the identical approach in another young patient with visceral heterotaxy. The ablation catheter is seen reaching the heart through a left sided hepatic vein. The pathway could be thereafter easily ablated. Figure 5 shows cessation of tachycardia during ablation with retrograde block in the pathway.

## Specialized Mapping Systems

### LocaLisa

The LocaLisa system locates diagnostic electrode catheters in three-dimensional space [13]. Analogous to the Frank lead system, three orthogonally positioned skin electrode pairs are used to send three small 1-mA currents through the thorax with slightly different frequencies of approximately 30kHz being used in each direction. The 30-kHz signals do not interfere with intracardiac EP signals. The posterior skin electrode of the system also serves as the return electrode for RF ablation. The mixture of 30-kHz signals recorded from each catheter electrode is digitally separated to measure the amplitude of each of the three frequency components. The three electrical field strengths are automatically calculated by using the difference in amplitude measured from neighbouring electrode pairs with a known interelectrode distance in at least three different spatial orientations of that dipole. The 3-D location in space of a given electrode is then calculated by dividing each of the three amplitudes by the corresponding electrical field strength. The electrode positions are averaged over 1 to 2 seconds to reduce the effect of cyclic heartbeat related variations. The system combines the advantages of ease of use and low cost, and does not require special catheters. It is applicable both for RF and cryoablation catheters. Several studies have demonstrated that the system considerably reduces fluoroscopic time [14]. However, unlike the other systems described next, LocaLisa does not allow activation or voltage mapping, and its use is limited when mapping complex reentrant circuits post-cardiac surgery [15]. With simpler tachyarrhythmia circuits, particularly AV nodal reentry, para-Hisian pathways and junctional ectopic tachycardia, continuous monitoring of the distal electrode of the ablation catheter and its relation to the His bundle during energy application increases the safety of RF ablation for these substrates [16-;18].

#### Example 3

**Figure 6:**
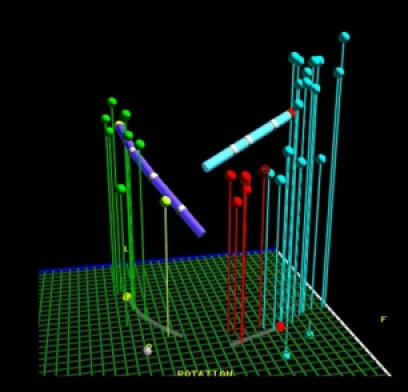
Slow pathway ablation utilising the LocaLisa navigation system. The coronary sinus catheter electrodes are shown in green, and the His bundle in blue. The red dots indicate ablation sites. When accelerated junctional rhythm occurs during RF application, atrial overdrive pacing is performed to confirm intact AV conduction.

Catheter ablation of the slow pathway in a young patient with AV nodal reentrant tachycardia. The location of the anatomical structures that form the corners of Koch's triangle are shown on the LocaLisa picture. The green dots represent the electrodes of a quadripolar catheter in the coronary sinus. The blue dots show locations at which His signals could be recorded. The red dots represnt individual RF lesions in the area of the slow pathway (Figure 6).

#### Example 4

**Figure 7:**
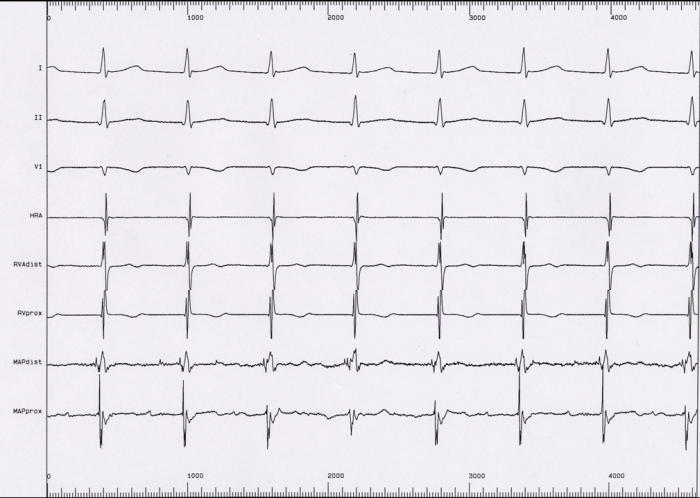
Congenital junctional ectopic tachycardia (see text for description)

**Figure 8:**
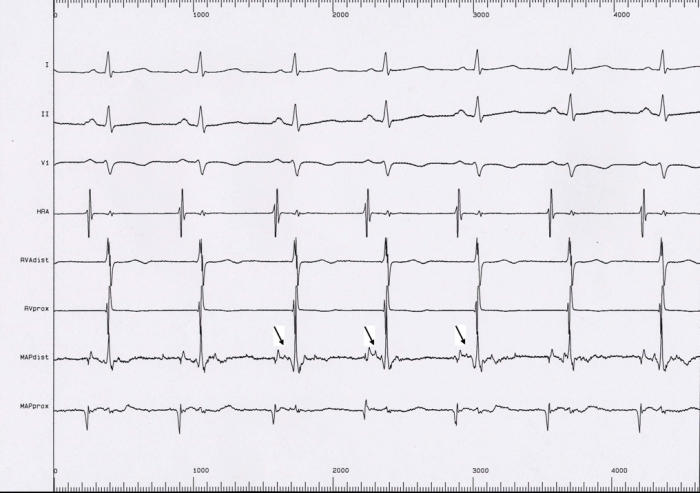
Ablation during sinus rhythm, targeting the site of earliest retrograde conduction during tachycardia. A discrete His potential can be seen.

**Figure 9:**
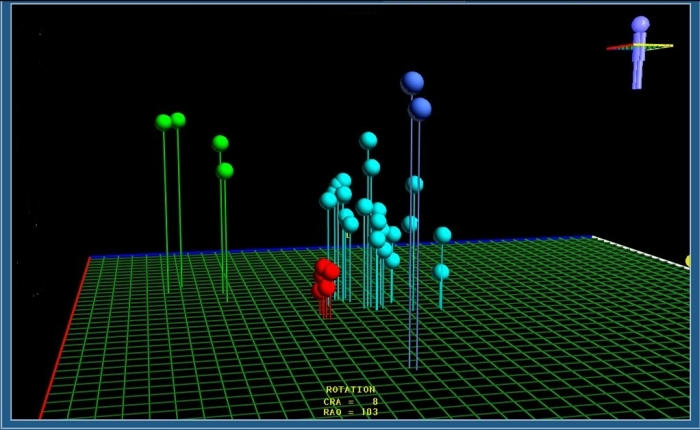
LocaLisa picture showing the proximity of the ablation sites to the His bundle.

Catheter ablation of congenital junctional ectopic tachycardia. The tachycardia electrogram shows 1:1 retrograde conduction, with near-simultaneous activation of the atrium and ventricle (Figure 7). Catheter ablation targeted the earliest site of retrograde conduction, and was performed during sinus rhythm; at the ablation site a discrete His electrogram can be seen (Figure 8). The accompanying LocaLisa picture (Figure 9) shows the proximity of the ablation site (red dots) to the His bundle (blue dots).

#### Example 5

**Figure 10:**
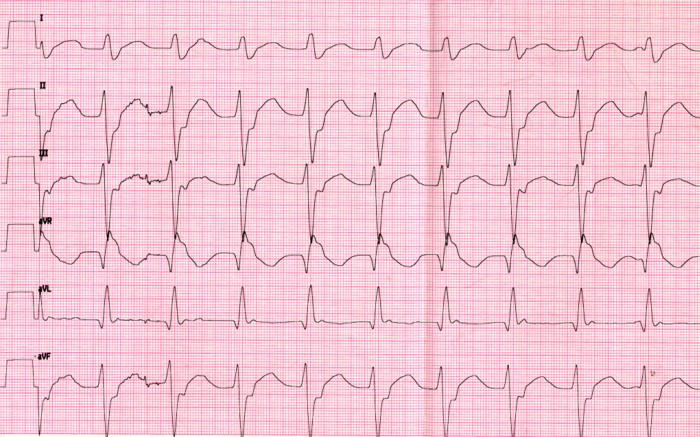

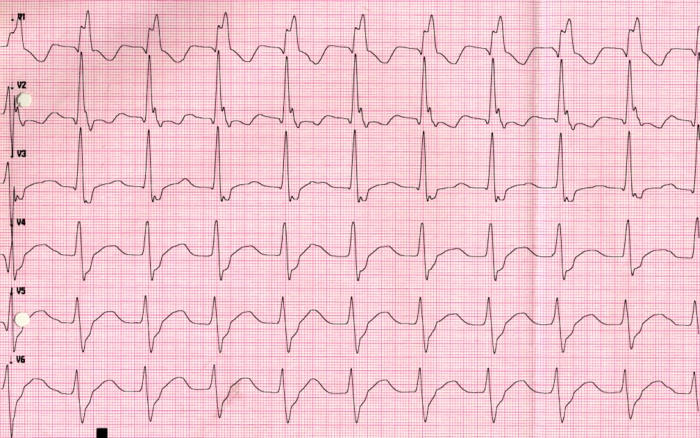
Reentrant posterior fascicular VT in a young patient.

**Figure 11:**
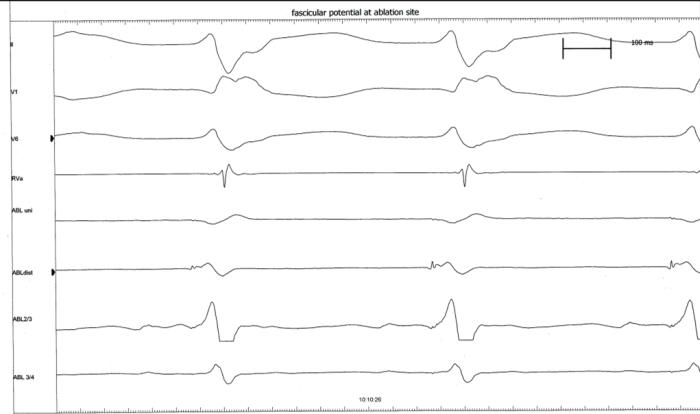
Intracardiac recording from an acutely successful ablation site, showing a discrete presystolic Purkinje potential.

**Figure 12:**
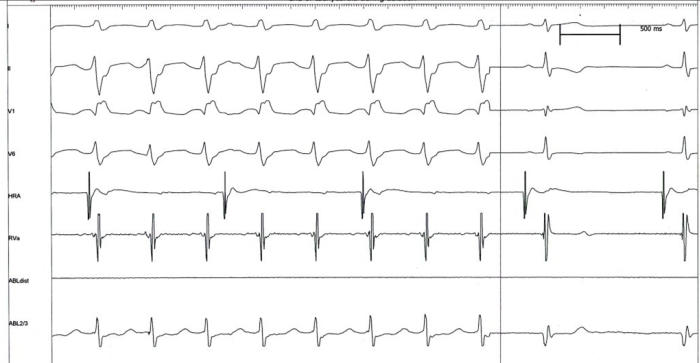
Termination of VT during RF catheter application at the above site. Tachyarrhythmia however recurred despite a second ablation targeting a distal Purkinje potential.

**Figure 13:**
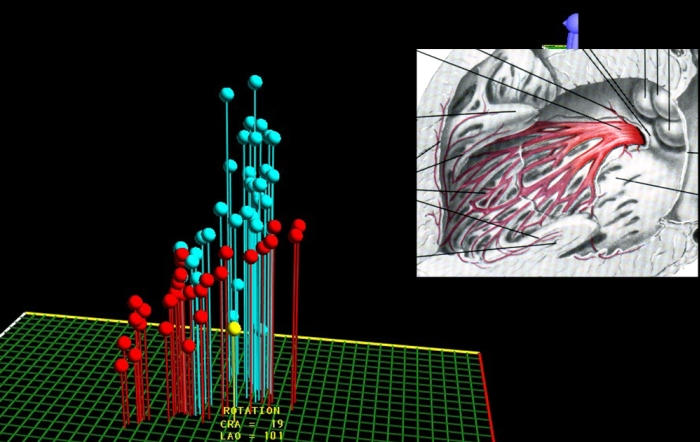
LocaLisa picture showing targeting of the proximal posterior fascicle during a third (and eventually successful) ablation. The blue dots show the proximal His bundle, and the red dots mark the ablation sites.

This 11 year old girl presented with incessant fascicular left ventricular tachycardia (Figure10 a and b). Two previous ablations targeting distal fascicular potentials (Figure 11 and Figure 12) had resulted in acute success, but with recurrence of tachycardia within a few weeks. At the third ablation, the posterior fascicle was interrupted proximally, with lasting success. The LocaLisa picture shows the His bundle (blue dots) recorded from the left ventricle, and the series of lesions made in close proximity to the bundle to produce posterior fascicular block (Figure 13).

### Electroanatomic mapping

The CARTO electroanatomic mapping system is a computerized system that displays both voltage and local electrogram timing in relation to a catheter tip (containing a location sensor) in 3-D space. Three ultra-low magnetic fields are emitted from a unit positioned under the patient, and data for amplitude, frequency and phase of the magnetic field are gathered and analysed by a processing unit to locate the catheter tip and its orientation. Three-dimensional maps are created point by point by first placing the catheter under fluoroscopic guidance in known anatomic locations that serve as landmarks. Other points of potential interest can then be obtained without the use of fluoroscopy. At each point a local electrogram can be recorded, and the obtained information can be displayed as a colour-coded voltage map, activation map and propagation map which is superimposed on the anatomic model of the chamber of interest. Isochronal maps display the local electrogram in relation to a stable reference electrode within an acquisition window which is set to the cycle length of the tachycardia. Such maps are useful when mapping focal tachyarrhythmias. The propagation map displays the wave of endocardial activation, and is particularly useful when studying macroreentrant circuits, such as intraatrial reentrant tachycardias following surgical repair of congenital heart defects [19]. The entire tachycardia circuit can be recreated by mapping sufficient points that together account for the cycle length of the tachycardia. In this way, areas of slow conduction (around scar tissue or anatomic barriers) and anatomical isthmuses that may be critical for tachycardia propagation can be visualized. Such mapping however should always be used in combination with standard EP techniques such as entrainment mapping to determine whether putative ablation sites are part of the reentry circuit. Occasionally however, entrainment pacing may result in a shift to another tachycardia circuit, or termination of tachycardia, particularly in patients with complex atrial anatomy following Fontan completion for single ventricle physiology. In such situations, Nakagawa et al have demonstrated that voltage mapping may be useful in identifying areas of low amplitude electrical activity, often running as channels between areas of scar tissue, which may be successfully targeted [20,;21].

Subsequent technical modifications of the CARTO system include the integration of MRI images with electroanatomic mapping (CARTOMERGE), and the use of multi-electrode mapping catheters (CARTO XP) to acquire multiple electroanatomic points simultaneously from each electrode, thereby shortening the potentially time-consuming process of constructing sequential point-by-point maps of a cardiac chamber.

#### Example 6

**Figure 14:**
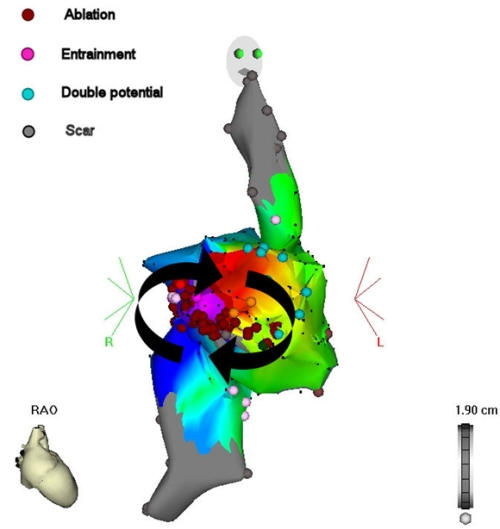
Cavo-tricuspid isthmus-dependent IART in a young man following a Mustard operation (see text).

This 15 year old boy presented with an intraatrial reentrant tachycardia after a previous Mustard operation. The IART was critically dependent on the cavo-tricuspid isthmus. The Carto image demonstrates the tachyarrhythmia circuit. To produce isthmus block however, ablation had to be performed both via the venous approach, and retrogradely via the femoral artery, to reach the part of the cavo-tricuspid isthmus which had been incorporated into the pulmonary venous atrium by the Mustard baffle (Figure 14). The same patient subsequently underwent stent implantation in the superior vena cava, to facilitate permanent transvenous pacemaker implantation for sinus node disease.

#### Example 7

**Figure 15:**
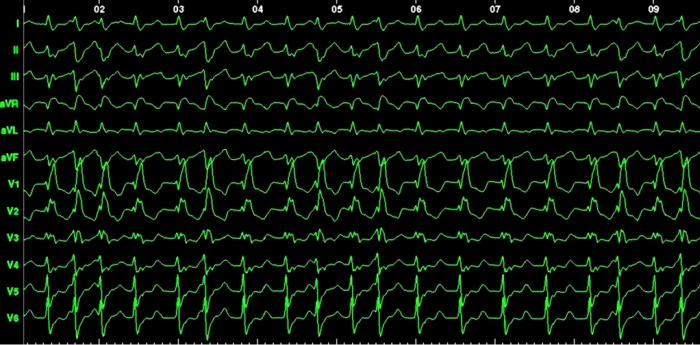
IART following a Rastelli procedure in a 42 year old man

**Figure 16-18:**
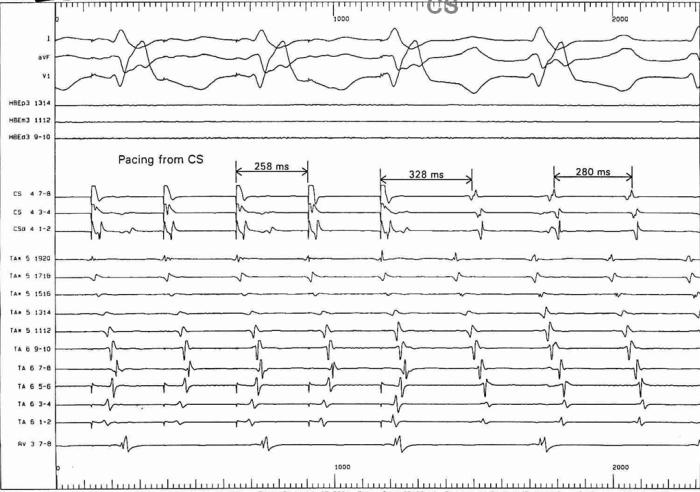

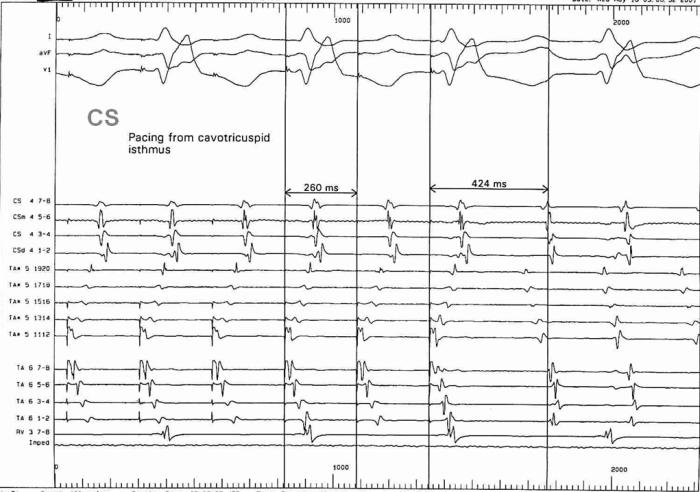

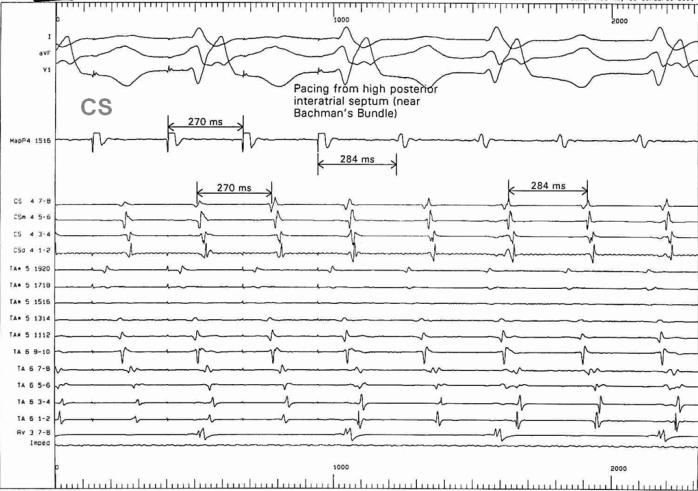
Entrainment pacing at various atrial sites. The closest match between the post-pacing interval and the spontaneous tachycardia cycle length is obtained during entrainment at a high posterior location in the right atrium (Figure 18).

**Figure 19-20:**
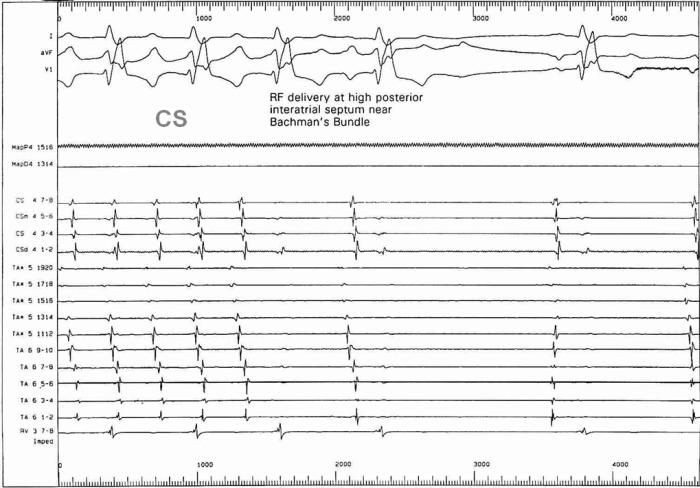

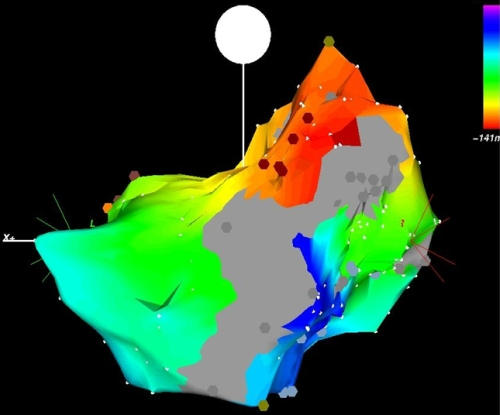
Termination of IART during ablation. The individual lesions are shown in dark red.

This 42 year old patient presented with refractory IART, having previously undergone surgical repair of double outlet right ventricle by a Rastelli procedure. Figure 15 shows his clinical arrhythmia. The IART could be entrained from several atrial sites, but the best match between spontaneous tachycardia cycle length and the post-pacing interval was obtained during entrainment pacing in the high posterior interatrial septum (near Bachman's bundle) (Figure 16 - 18). RF ablation at this site resulted in termination of tachycardia (Figure 19). The accompanying Carto map is shown (Figure 20).

### Noncontact Mapping

The EnSite mapping system uses a 9F catheter equipped with an inflatable balloon with a multielectrode array for recording intracardiac signals within a cardiac chamber. The boundaries of the chamber are defined using a roving catheter that emits a 5.68 kHz low current locator signal. When defining chamber geometry, each passage of the roving catheter to a new location in a given direction from the multielectrode array defines the endocardial boundary, thus building a 3-D reconstruction of the chamber. Once the chamber boundaries have been defined, a potential voltage map can be created from a single cardiac cycle. A computer workstation employs an inverse solution to the LaPlace equation to process the amplified far-field signals from the noncontact catheter and creates a 3-D endocardial potential map. Upto 3000 virtual unipolar electrograms can be calculated and displayed onto the computer-generated 3-D image of the endocardial surface. While this technology is potentially useful in mapping arrhythmias that are not haemodynamically well tolerated, using only a few arrhythmia beats, there are important limitations to the EnSite system in paediatric practice [22]. First, resolution of the system is good for distances of upto 3.5 cm, so that larger chambers may nor be reliably mapped. Secondly, it may not be possible to safely deploy the balloon catheter within chambers of critical importance to the tachycardia circuit, for example within the pulmonary venous atrium in patients who have undergone a Mustard or Senning procedure for transposition of the great arteries.

The EnSite system can also be used as a pure navigation tool, without the use of the noncontact multielectrode array. A surface-based system is then used to define cardiac chamber anatomy and to continuously visualize catheter position (EnSite NavX). Upto 8 catheters and 64 electrodes can be visualized in 3-D space. In addition to continuously visualizing catheter position in any of the cardiac chambers, lesion markers can also be tagged to the 3-D reconstruction, to visualize continuity of ablation lines.

#### Example 8

The video clip (Figure 21) shows the right ventricular activation sequence is shown in a young teenager with a focal VT arising from the right ventricular outflow tract. This was successfully ablated.

## Special considerations and risks in paediatric ablation

### 1. Increase of lesion size with age

Saul et al demonstrated in an infant lamb model that RF lesions tended to enlarge with increased duration of follow-up, and were associated with fibrous tissue invasion of normal myocardium [23]. It was postulated that these findings may have implications for RF ablation in human infants, with regard to production of new arrhythmogenic substrates. Similar findings however were not shown in a pig model [24]. From clinical follow-up data, there have been no reports of later arrhythmias arising from a site where RF ablation was performed in infancy. Echocardiographic follow-up data also suggest that RF lesions appear to shrink with time [25]. This is however an issue where long-term follow-up into adult life may be required.

### 2. AV block

The risk of AV block is obviously higher in younger patients, when modifying substrates such as AV nodal reentrant tachycardia or ablating para-Hisian accessory pathways. To some extent such complications are also related to operator experience, and RF ablation in infancy is generally to be avoided except in instances of drug-resistant incessant arrhythmias associated with ventricular dysfunction or cardiomyopathy. The use of cryoablation should also be considered in selected instances (see below).

### 3. Coronary artery lesions

There have been several reports of coronary artery stenoses or acute occlusion of a coronary artery in close proximity to a site of RF lesion application These have been seen following ablation of accessory pathways in all locations, and affecting each of the three main coronary arteries [26,27]. Again, the relative risks and benefits of ablation should be carefully considered in every young patient, and it is important to be aware of this complication. We do not routinely perform coronary artery angiography following every RF ablation procedure in young patients, although this has been suggested.

## Cryoablation

Although RF is currently the energy source of choice for catheter ablation in children, there are some special situations where an alternative energy source may play a role. The role of cryoablation in paediatric EP practice is now well established. Potential advantages of cryoablation include the ablitiy to produce reversible lesions during cryomapping (achieving catheter tip temperatures of between -20ºC and -30ºC for periods of upto 60 seconds, with complete reversibility of the effects of energy application on the target tissue when the catheter tip is allowed to rewarm), increased catheter stability during application of a permanent cryolesion (the procedure is virtually hands-off during the 4 minutes required to achieve a therapeutic cryolesion), the decreased incidence of thrombus formation, and the ability to ablate within venous structures or in close proximity to a coronary artery. Even during application of a permanent lesion (typically at temperatures of around -75ºC) if an undesired effect is observed (such as complete AV block), prompt termination of cryoablation application usually results in complete recovery of AV conduction. For all these reasons, cryoablation may be favoured when ablating AV nodal reentrant tachyarrhythmia or para-Hisian accessory pathways in small children [28,29]. Early results however do suggest a higher recurrence rate of arrhythmia in comparison with standard RF ablation [30].

## Irrigated or cooled-tip RF ablation

During temperature-controlled RF ablation, both the catheter tip temperature and tissue temperature are affected by electrode to tissue contact and the cooling effects of circulating blood. With good electrode contact and low cooling of the catheter tip, the target temperature can be achieved with low power output, resulting in a small lesion size. An irrigated tip electrode decreases the electrode-myocardial interface temperature and allows for a larger amount of RF current to be delivered before heating of the tissue. Cooled tip ablation therefore allows both a higher power output and a longer duration of RF energy delivery, resulting in a larger and deeper lesion. These advantages are potentially of benefit when ablating some ventricular tachyarrhythmias, intraatrial reentrant arrhythmias following a Fontan completion (when the right atrium may be abnormally thickened), or even for some accessory atrioventricular connections which may be epicardially located [31]. Occasionally, cooled tip ablation may also be used to reduce (debulk) intracardiac muscle or tumour masses [32].

## Conclusions

Invasive EP studies and catheter ablation techniques have seen tremendous progress in the last 20 years, and their application even in small infants and children is considered standard therapy. With increased operator experience, improvements in available catheter and mapping technology, the safety and success rates of ablation procedures in children has also improved. The use of additional energy sources (cryoablation) or irrigated/cooled tip catheters have also increased both the safety and acute success of ablation.

Recurrence of arrhythmia after an initially successful ablation is commoner in children when compared with adults [33]. Especially when dealing with post-operative arrhythmia substrates, it is important to bear in mind that recurrences are commoner, that newer arrhythmia substrates may develop in association with chronic changes in the myocardium as a result of increasing pressure or volume overload, and that arrhythmia cure may be only one part of a global strategy for optimising outcome, which may include other measures such as long-term pharmacologic therapy, catheter intervention procedures for residual lesions, or reoperation for residual or progressive haemodynamic sequelae [34].

## Figures and Tables

**Figure 21:** Ensite RV activation sequence in a young man with idiopathic right ventricular outflow tract tachycardia undergoing successful RF ablation. Click here to view the movie
